# The Dipeptide Pro-Gly Promotes IGF-1 Expression and Secretion in HepG2 and Female Mice via PepT1-JAK2/STAT5 Pathway

**DOI:** 10.3389/fendo.2018.00424

**Published:** 2018-07-26

**Authors:** Mengyuan Zhang, Jingren Xu, Tao Wang, Xiaojuan Wan, Fenglin Zhang, Lina Wang, Xiaotong Zhu, Ping Gao, Gang Shu, Qingyan Jiang, Songbo Wang

**Affiliations:** ^1^Guangdong Provincial Key Laboratory of Animal Nutrition Control, College of Animal Science, South China Agricultural University, Guangzhou, China; ^2^National Engineering Research Center for Breeding Swine Industry and ALLTECH-SCAU Animal Nutrition Control Research Alliance, South China Agricultural University, Guangzhou, China

**Keywords:** dipeptide, Pro-Gly, IGF-1, HepG2, female mice, JAK2/STAT5, PepT1

## Abstract

It has been shown that IGF-1 secretion is influenced by dietary protein or amino acid. However, whether the dipeptides elicit regulatory effects on IGF-1 secretion remains largely unclear. Thus, this study aimed to investigate the effects of the dipeptide Pro-Gly on IGF-1 expression and secretion in HepG2 cells and mice, and explore the underlying mechanisms. The *in vitro* results indicated that Pro-Gly, but not Pro plus Gly, promoted the expression and secretion of IGF-1 in HepG2. Meanwhile, the expression of the peptide transporter 1 (PepT1) was elevated by Pro-Gly, whereas knockdown of PepT1 with siRNA eliminated the increase of IGF-1 expression induced by Pro-Gly. In addition, Pro-Gly activated JAK2/STAT5 signaling pathway in a PepT1-dependent manner. Furthermore, Pro-Gly enhanced the interaction between JAK2 and STAT5, and the translocation of phospho-STAT5 to nuclei. Moreover, inhibition of JAK2/STAT5 blocked the promotive effect of Pro-Gly on IGF-1 expression and secretion. In agreement with the *in vitro* results, the *in vivo* findings demonstrated that Pro-Gly, but not Pro plus Gly, stimulated the expression and secretion of IGF-1 and activated JAK2/STAT5 signaling pathway in the liver of mice injected with Pro-Gly or Pro+Gly acutely or chronically. Besides, acute injection of JAK2/STAT5 inhibitor abolished the elevation of IGF-1 expression and secretion induced by Pro-Gly in mice. Collectively, these findings suggested that the dipeptide Pro-Gly promoted IGF-1 expression and secretion in HepG2 cells and mice by activating JAK2/STAT5 signaling pathway through PepT1. These data provided new insights to the regulation of IGF-1 expression and secretion by the dipeptides.

## Introduction

Insulin-like growth factor-1 (IGF-1) is a 70-amino acid hormone with effects on almost every tissue and organ (1–3), playing an essential role in growth (4, 5), metabolism (6, 7), and survival (8, 9). Thus, the regulation of IGF-1 production is of great significance to the normal physiological process of the diverse organisms. The circulating IGF-1 is primarily produced by liver following growth hormone (GH) endocrine stimulus (3, 10). The binding of circulating GH leads to dimerization of its receptor and activates Janus kinase 2 (JAK2). JAK2 then recruits and phosphorylates signal transducer and activator of transcription (STAT) 5, leading to its dimerization and translocation to the nucleus, where it binds to specific regulatory elements of IGF-1 and stimulates the production of hepatic IGF-1 (11–13).

Besides GH, the level of IGF-1 is also regulated by nutrition such as dietary proteins (14–16) or amino acids (17–19). The dipeptides are one of the products resulting from the digestion of dietary proteins, which can be absorbed by the intestine and delivered to the liver via hepatic portal vein. It has been demonstrated that the dipeptides are actively transported across membranes as an efficient route for dietary protein absorption via a proton-coupled peptide transporter 1 (PepT1) (20), which is present in the various tissues such as intestine (21), kidney (22), pancreas, bile duct and liver (23). Besides the important nutritional value, the dipeptides have been implicated to exert various physiological functions. It has been reported that Ala-Gln and Glu-Phe is involved in ameliorating peritoneal fibrosis (24) and attenuating lipogenesis in hepatocytes (25), respectively. The dipeptide Phe-Cys exerts antioxidant function in hepatocytes (26), while the dipeptides Gly-Gly and Arg-Arg have anti-inflammatory effects via PepT1 (27, 28). The dipeptide Trp-His has been shown to elicit anti-atherosclerotic effects in rats (29), and Hyp-Gly promotes C2C12 myoblast differentiation and myotube hypertrophy (30).

The dipeptide prolyl-glycine (Pro-Gly) was identified as one of serum prolyl dipeptides and its concentration in normal human serum could be effectively detected (31). Meanwhile, the major food-derived elastin peptide Pro-Gly is present in human plasma after oral ingestion of elastin hydrolysate (32). In addition, Pro-Gly significantly enhanced elastin synthesis of normal human dermal fibroblasts (NHDF) without affecting the rate of cell proliferation (32). Furthermore, Noopept (ethyl ester of N-phenylacetyl-L-prolylglycine), Pro-Gly-containing dipeptide, increased the DNA-binding activity of hypoxia-inducible factor 1 (HIF-1) in HEK293 cells (33). These results suggested the bioactive effects of the dipeptide Pro-Gly. However, the effect of the dipeptide Pro-Gly on regulating IGF-1 secretion in HepG2 cells and mice remains unknown.

The purpose of this study was to determine whether the prolyl dipeptide Pro-Gly had potential effects on IGF-1 secretion in HepG2 cells and C57BL/6J mice. In addition, we sought to explore the possible mechanisms underlying in this process, including the contribution of PepT1 and JAK2/STAT5 signaling pathway. Here, we provided evidence that the dipeptide Pro-Gly promoted IGF-1 expression and secretion in HepG2 cells and mice by activating JAK2/STAT5 signaling pathway through PepT1.

## Materials and methods

### Chemicals and antibodies

The dipeptide Pro-Gly (P0880), Pro (P5607), Gly (V900144) were purchased from Sigma-Aldrich (St. Louis, MO, USA). JAK2 inhibitor AZD1480 (S2162) were purchased from Selleck (Shanghai, China), Dulbecco's modified Eagle's medium (DMEM), HepatoZYME-SFM and fetal bovine serum (FBS) were purchased from Gibco BRL (Carlsbad, CA). Rabbit polyclonal antibodies against β-actin (Cat# bs-0061R, RRID: AB_10855480), β-tubulin (Cat# bs-4511R, RRID: AB_11114300), and 4hosphor-JAK2 (Tyr1007/Tyr1008) (Cat# bs-2485R, RRID: AB_10855229) were purchased from Bioss (Beijing, China). Rabbit monoclonal antibody against 4hosphor-STAT5 (Tyr694) (Cat# AB32364, RRID: AB_778105) was purchased from Abcam (Cambridge, MA, USA). Rabbit monoclonal antibodies against JAK2 (Cat# 3230, RRID: AB_2128522), and polyclonal antibodies against STAT5 (Cat# 9363S, RRID: AB_10693321) were purchased from Cell Signaling Technology Inc (Beverly, MA, USA). Rabbit polyclonal antibody against IGF-1 (Cat# 20214-1-AP, RRID: AB_10666736) was purchased from Proteintech Inc (Rosemont, CA, USA). The related secondary antibodies were purchased from Bioworld (Nanjing, China).

### Cell culture and treatment

HepG2 cells were seeded in a 6-well plate at density of 4 × 10^6^ cells and cultured in DMEM supplemented with 10% FBS, 100 U/ml penicillin, and 100 mg/ml streptomycin in a humidified cell incubator with 5% CO_2_ at 37°C. When the cells reached about 80% confluence, the culture medium was replaced with above mentioned medium containing various treatments as follows: (1) Pro-Gly (0.2, 0.5 and 1 mM), (2) Pro-Gly (0.5 mM) or Pro+Gly (0.5 mM), (3) Pro-Gly (0.5 mM) and/or AZD1480 (1 μM). After 6 or 24 h, the cell culture supernatant was collected to determine the secretion of IGF-1. At the same time, the cells were collected to detect the IGF-1 expression and the related intracellular signaling pathways.

### Animals and *In Vivo* study

The C57BL/6J female mice were purchased from Guangdong Medical Laboratory Animal Center [permission number: SYXK (Guangdong) 2014-0136] and housed in environmentally controlled rooms on a 12-h light-dark cycle with free access to food and water. All animal experiments and care procedures were performed according to the guidelines for the care and use of animals approved by The Animal Ethics Committee of South China Agricultural University. There are three injection experiments:

Experiments 1 and 2: injection Pro-Gly or Pro plus Gly

For experiment 1 (acute injection), 18 6-week-old mice were randomly divided into three groups: Control, Pro-Gly, and Pro+Gly. The mice in the three groups were intraperitoneal injected with physiological saline (Control), Pro-Gly (100 mg/kg), or Pro (58 mg/kg) plus Gly (38 mg/kg) (Pro+Gly) in a volume of 100 μL at 6 p.m., respectively. After 1 h, the mice were sacrificed by carbon dioxide anesthesia for blood and liver samples collection.

For experiment 2 (chronic injection), 30 4-week-old mice were randomly divided into three groups: Control, Pro-Gly, and Pro+Gly. Each group received physiological saline (Control), Pro-Gly (150 mg/kg), or Pro (87 mg/kg) plus Gly (57 mg/kg) (Pro+Gly) intraperitoneal injection once every other day in a volume of 100 μL at 6 p.m. for 35 days, respectively. On day 35, the mice were killed alternately in 3 groups starting from 9 a.m. and the blood and liver samples were harvested.

Experiment 3: blockade of Jak2 *in vivo*

Twenty-eight mice (6 weeks age) were randomly divided into four groups: Control, Pro-Gly, AZD1480, and AZD1480+Pro-Gly, which were intraperitoneal injected with physiological saline, physiological saline, AZD1480 (30 mg/kg), and AZD1480 (30 mg/kg) in a volume of 100 μL at 4 p.m., respectively. Then, at 6 p.m., the mice in Control, Pro-Gly, AZD1480, and AZD1480+Pro-Gly groups were intraperitoneal injected with physiological saline, Pro-Gly (100 mg/kg), physiological saline, and Pro-Gly (100 mg/kg) in a volume of 100 μL, respectively. After 1 h, the mice were sacrificed to collect blood and liver samples for further analysis.

The blood was collected and incubated at 37°C for 1 h and then centrifuged at 1,500 g for 20 min. Then the serum was collected and stored at −20°C for further determination of serum IGF-1 level. The liver was isolated and frozen in liquid nitrogen and then stored at −80°C until further analyses.

### Real-time quantitative PCR

The *IGF-1* mRNA levels were examined by real-time quantitative PCR as we previously described (34). Briefly, total RNA was extracted by using TRIzol reagent (Invitrogen, Carlsbad, CA, USA) according to the manufacturer's protocol and cDNA was synthesized from 2 μg of total RNA by the M-MLV Reverse Transcriptase (Promega, Madison, WI, USA) and random primers oligo-dT18 according to the manufacturer's instructions. Human *GAPDH* and mice β*-actin* was used as a candidate housekeeping gene. Real-time quantitative PCR was carried out in Mx3005p instrument (Stratagene, La Jolla, CA, USA) by using SYBR Green Real-time PCR Master Mix reagents (Toyobo Co., Ltd., Osaka, Japan) and both sense and antisense primers (200 nM for each gene). Primer sequences (with their respective PCR fragment lengths) were shown in Table 1.

**Table 1:** The primer sequences used for real-time quantitative PCR.

**Gene**	**Primer sequences (5′-3′)**	**Amplification length (bp)**
Homo *GAPDH*	F: ACGCATTTGGTCGTATTGGG	231
	R: TGATTTTGGAGGGATCTCGC	
Homo *IGF-1*	F: CATGTCCTCCTCGCATCTCT	212
	R: AGCAGCACTCATCCACGATA	
Homo *PepT1*	F: GGCCAGTTCAGCAAACAGTG	237
	R: GTCCATCCTCCACTTGCCTC	
Mus *β-actin*	F: GGTCATCACTATTGGCAACGAG	142
	R: GAGGTCTTTACGGATGTCAACG	
Mus *IGF-1*	F: CTGGACCAGAGACCCTTTGC	269
	R: GGACGGGGACTTCTGAGTCTT	
Mus *PepT1*	F: CACAATAAACACCACGGCGG	80
	R: TATACGCGCTGCCAAAGTCA	

### Western blot assay

**Figure 1:**
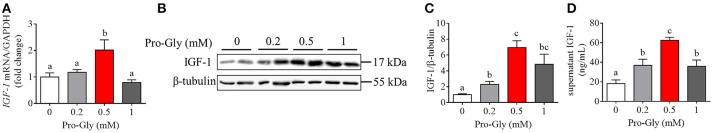
Pro-Gly promoted the expression and secretion of IGF-1 in the HepG2 cells. **(A)** Relative *IGF-1* mRNA level in the HepG2 cells after 24 h incubation with various concentrations (0.2, 0.5, and 1 mM) of Pro-Gly. **(B)** Western blot analysis of prepro IGF-1 in the HepG2 cells after 24 h incubation with various concentrations of Pro-Gly. β-tubulin was used as loading control. The panels shown are the representative bands of 3 independent experiments with 6 replicates. **(C)** Mean ± SEM of immunoblotting bands of prepro IGF-1 (*n* = 6). **(D)** Effect of Pro-Gly on the IGF-1 content in the supernatant of HepG2 cells after 24 h incubation. Bars that do not share the same letter are significantly different (*P* < 0.05).

**Figure 2:**
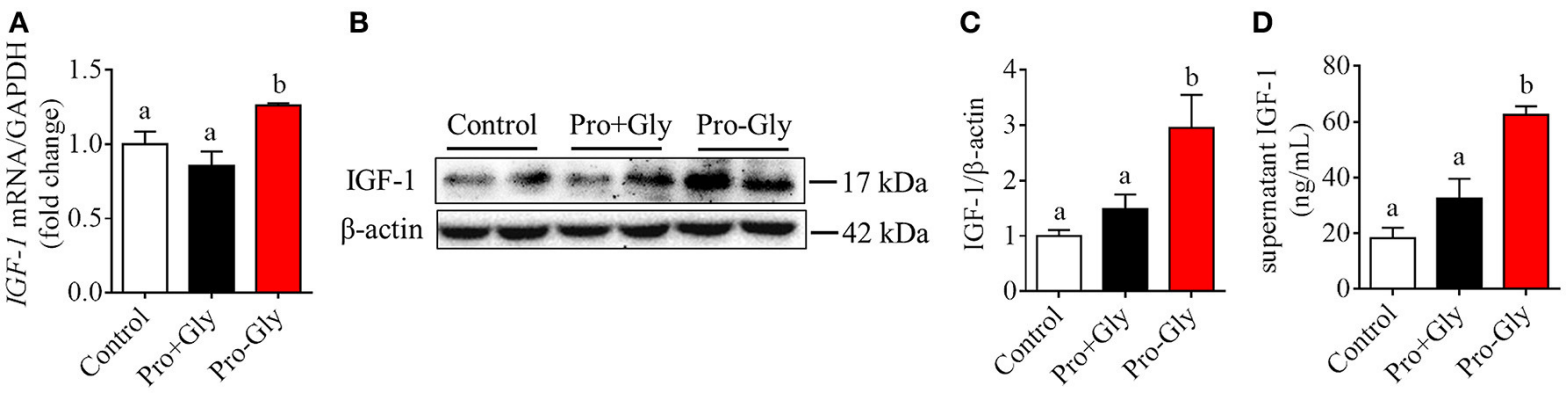
Pro-Gly, but not Pro plus Gly (Pro+Gly), promoted the expression and secretion of IGF-1 in the HepG2 cells. **(A)** Relative *IGF-1* mRNA level in the HepG2 cells after 24 h incubation with 0.5 mM Pro-Gly or Pro+Gly. **(B)** Western blot analysis of prepro IGF-1 in the HepG2 cells after 24 h incubation with 0.5 mM Pro-Gly or Pro+Gly. β-actin was used as loading control. The panels shown are the representative bands of 3 independent experiments with 6 replicates. **(C)** Mean ± SEM of immunoblotting bands of prepro IGF-1 (*n* = 6). **(D)** Effect of 0.5 mM Pro-Gly or Pro+Gly on the IGF-1 content in the supernatant of HepG2 cells after 24 h incubation. Bars that do not share the same letter are significantly different (*P* < 0.05).

**Figure 3:**
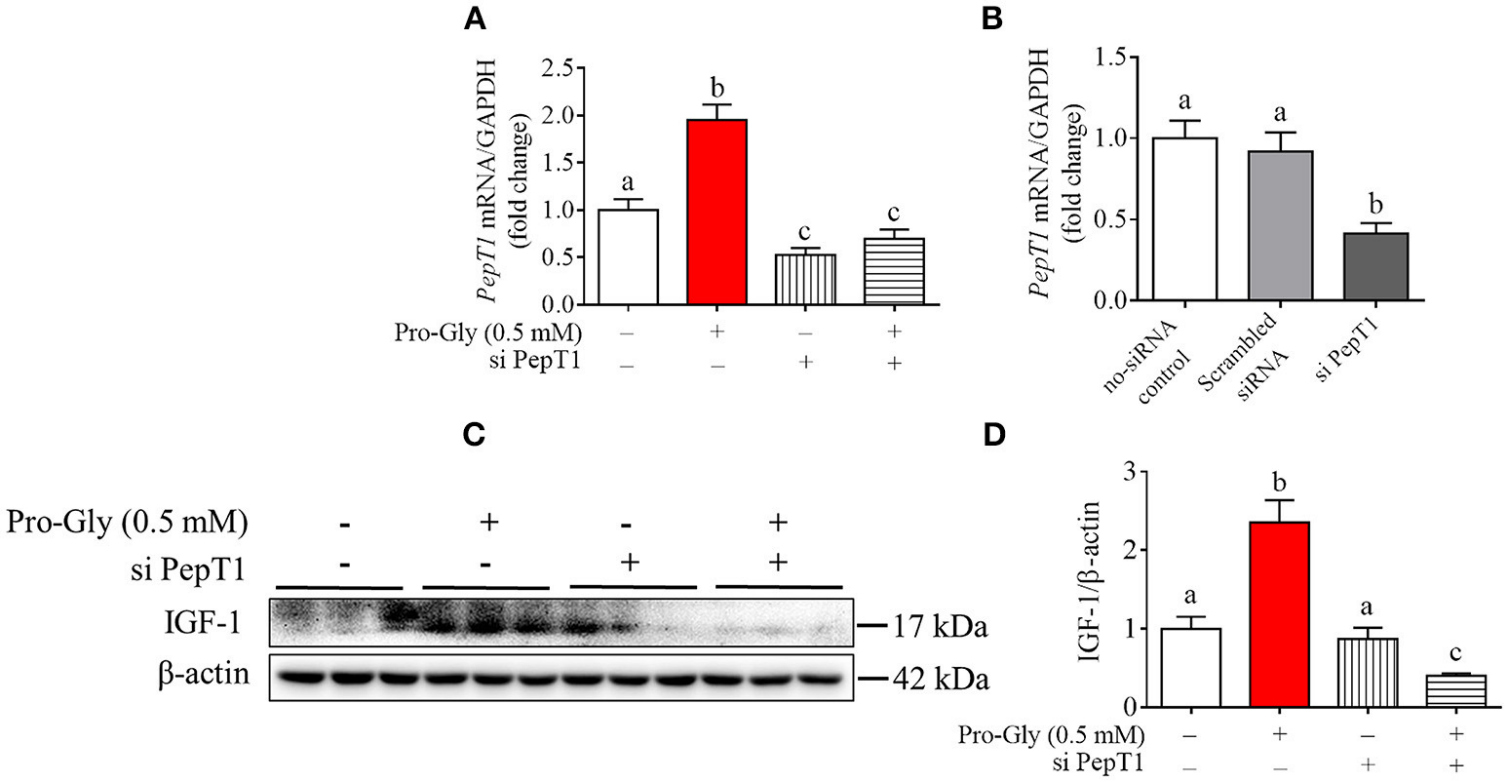
Knockdown of PepT1 eliminated the increase of IGF-1 expression induced by Pro-Gly in the HepG2 cells. **(A)** Relative *PepT1* mRNA level in HepG2 cells after 24 h incubation in the presence of Pro-Gly and/or PepT1 siRNA (si PePT1). GAPDH was used as the housekeeping gene. **(B)** The relative *PepT1* mRNA level in response to PepT1 siRNA in HepG2 cells after 6 h incubation. GAPDH was used as the housekeeping gene. **(C)** Western blot analysis of prepro IGF-1 in the HepG2 cells after 24 h incubation in the presence of Pro-Gly (0.5 mM) and/or PepT1 siRNA. β-actin was used as loading control. The panels shown are the representative bands of 3 independent experiments with 6 replicates. **(D)** Mean ± SEM of immunoblotting bands of prepro IGF-1 (*n* = 6). Bars that do not share the same letter are significantly different (*P* < 0.05).

**Figure 4:**
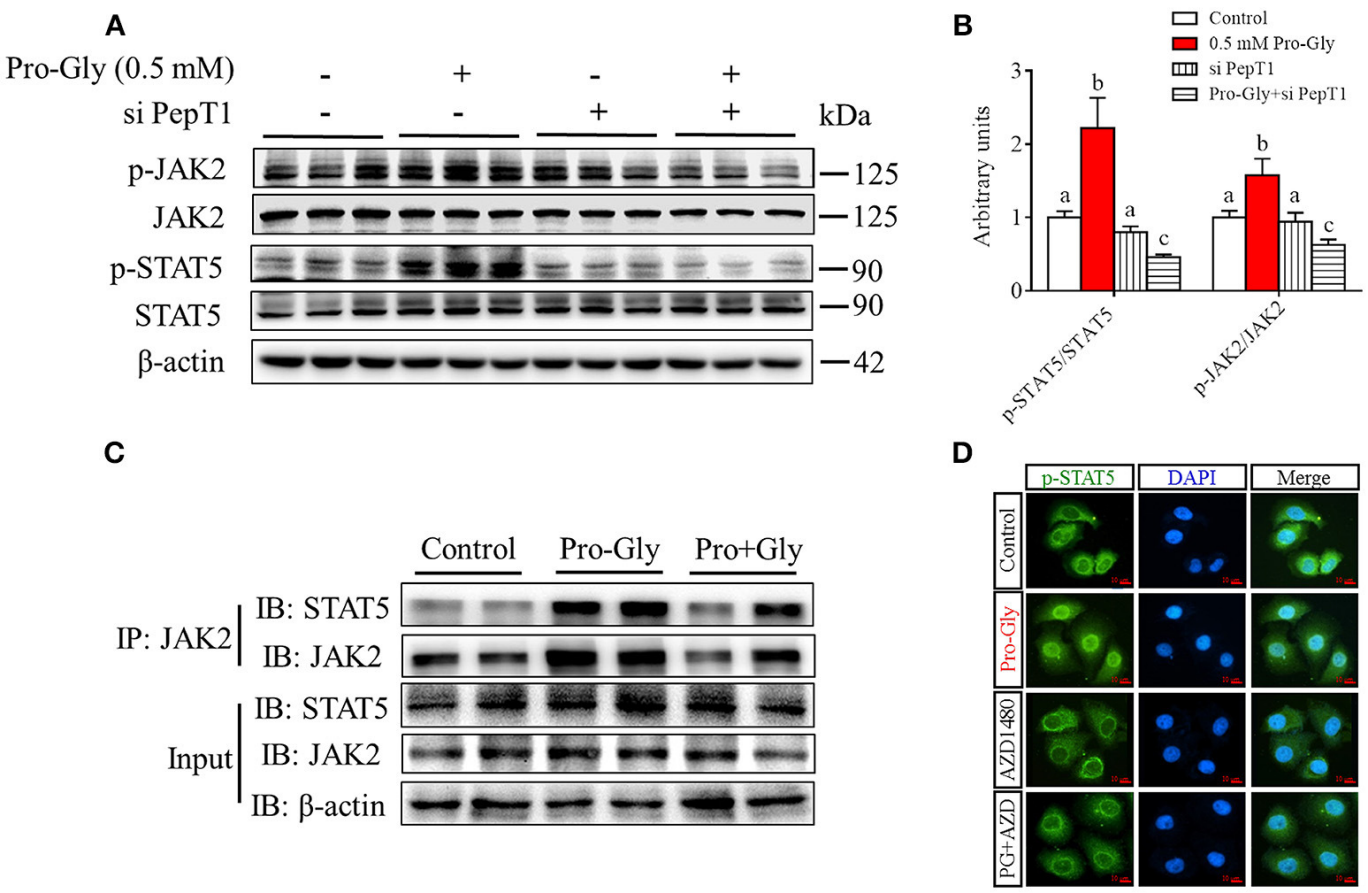
Pro-Gly activated JAK2/STAT5 signaling pathway in a PepT1-dependent manner. **(A)** Western blot analysis of phospho-JAK2 (p-JAK2), JAK2, phospho-STAT5 (p-STAT5), and STAT5 in HepG2 cells after 24 h incubation in the presence of Pro-Gly (0.5 mM) and/or PepT1 siRNA. β-actin was used as loading control. The panels shown are the representative bands of 3 independent experiments with 6 replicates. **(B)** Mean ± SEM of immunoblotting bands of p-JAK2/JAK2 and p-STAT5/STAT5 (*n* = 6). The intensities of the bands were expressed as the arbitrary units. Bars that do not share the same letter are significantly different (*P* < 0.05). **(C)** Interaction (binding) between JAK2 and STAT5 detected by co-IP. HepG2 cells were exposed to 0.5 mM Pro-Gly or 0.5 mM Pro+Gly for 24 h. **(D)** HepG2 cells were incubated in the presence of Pro-Gly (0.5 mM) and/or AZD1480 (1 μM) for 6 h and phospho-STAT5 translocation to nuclei was detected by ICC. Scale bar, 10 μm. The IP and ICC experiments were conducted independently for 3 times, with 3 replicates each time.

**Figure 5:**
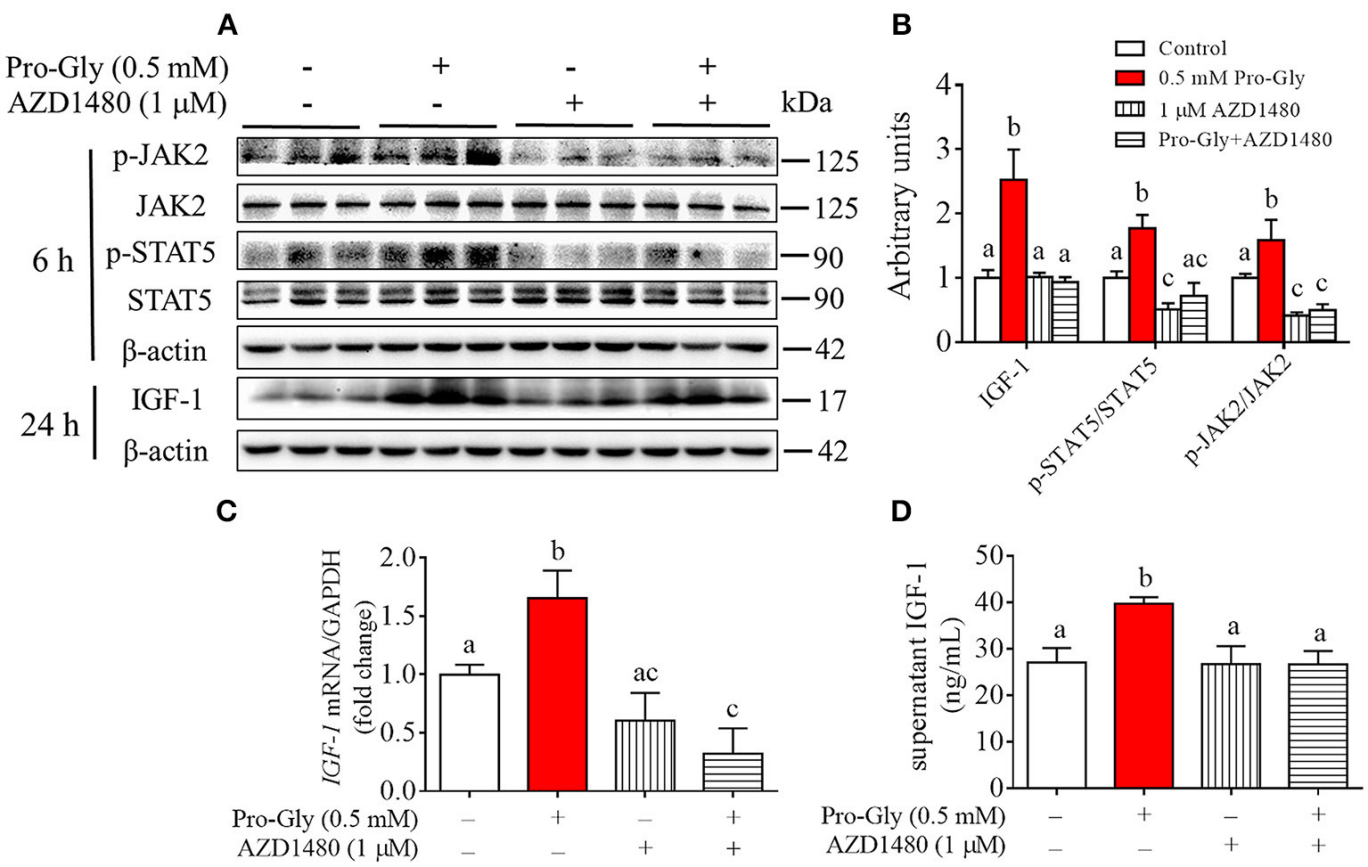
Inhibition of JAK2/STAT5 signaling pathway blocked the promotive effect of Pro-Gly on IGF-1 expression and secretion in the HepG2 cells. **(A)** Western blot analysis of phospho-JAK2 (p-JAK2), JAK2, phospho-STAT5 (p-STAT5), STAT5 and prepro IGF-1 in HepG2 cells after 6 h or 24 h incubation in the presence of Pro-Gly (0.5 mM) and/or AZD1480 (1 μM), respectively. β-actin was used as loading control. The panels shown are the representative bands of 3 independent experiments with 6 replicates. **(B)** Mean ± SEM of immunoblotting bands of prepro IGF-1, p-JAK2/JAK2 and p-STAT5/STAT5 (*n* = 6). The intensities of the bands were expressed as the arbitrary units. **(C)** *IGF-1* mRNA level in HepG2 cells after 24 h incubation in the presence of Pro-Gly (0.5 mM) and/or AZD1480 (1 μM) (*n* = 6). GAPDH was used as housekeeping gene. **(D)** Effects of Pro-Gly (0.5 mM) and/or AZD1480 (1 μM) on IGF-1 levels in the supernatant of HepG2 cells (*n* = 6). Bars that do not share the same letter are significantly different (*P* < 0.05).

**Figure 6:**
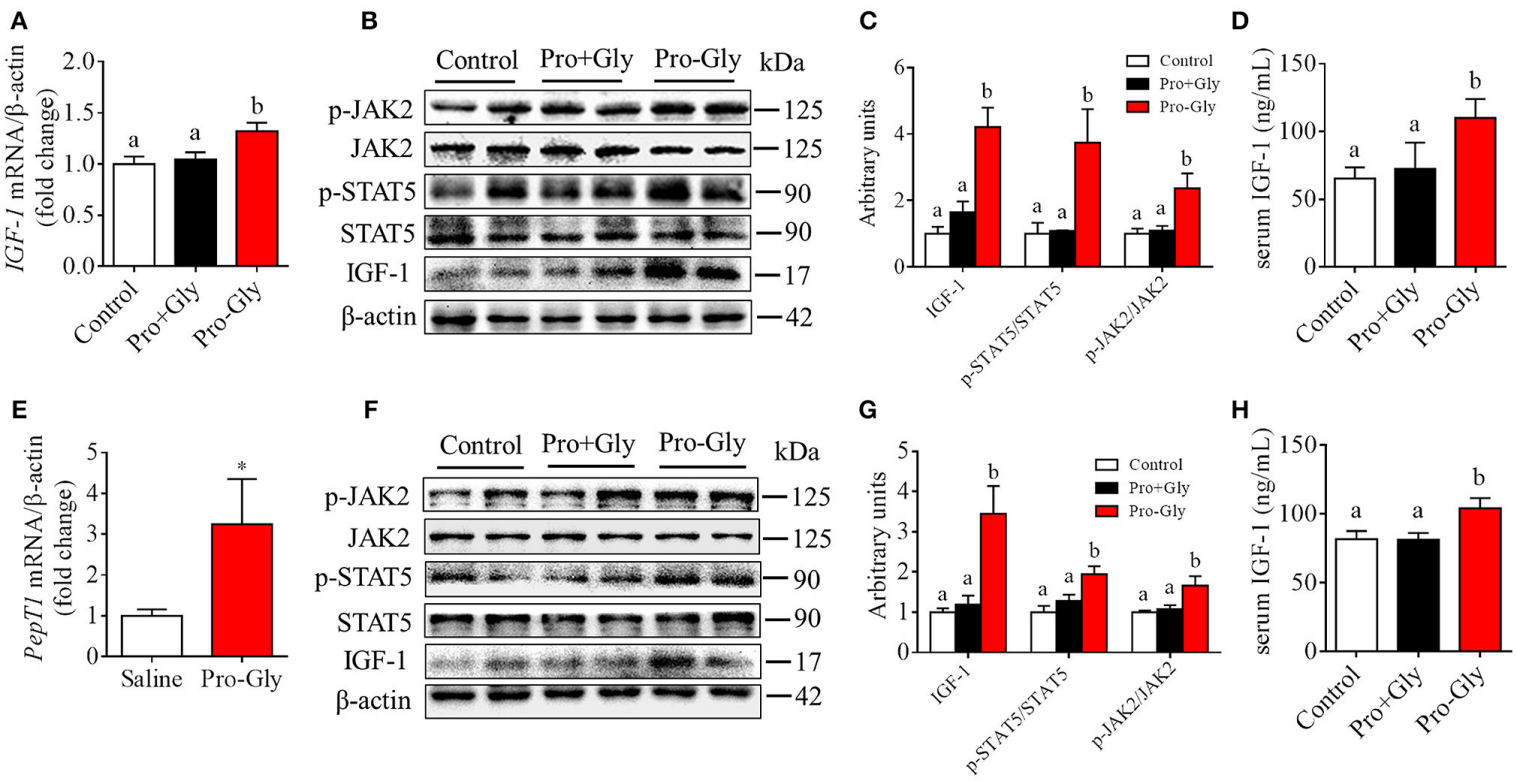
Acute or chronic injection of Pro-Gly, but not Pro plus Gly (Pro+Gly), stimulated IGF-1 expression and secretion in mice. **(A–D)** The 18 6-week-old female mice were intraperitoneal injected with physiological saline (Control, *n* = 6), Pro-Gly (100 mg/kg, *n* = 6), or Pro (58 mg/kg) plus Gly (38 mg/kg) (Pro+Gly, *n* = 6) for 1 h. Effects of acute injection of Pro-Gly or Pro+Gly on *IGF-1* mRNA level **(A)**, prepro IGF-1 protein expression and activation of JAK2/STAT5 signaling pathway **(B,C)** in mice liver and serum level of IGF-1 **(D)**. β-actin was used as loading control. The intensities of the bands were expressed as the arbitrary units. **(E–H)** The 30 4-week-old female mice were intraperitoneal injected with physiological saline (Control, *n* = 10), Pro-Gly (150 mg/kg, *n* = 10), or Pro (87 mg/kg) plus Gly (57 mg/kg) (Pro+Gly, *n* = 10) every other day for 35 days. Effects of chronic injection of Pro-Gly or Pro+Gly on *PepT1* mRNA level **(E)**, prepro IGF-1 protein expression and activation of JAK2/STAT5 signaling pathway **(F,G)** in mice liver and serum level of IGF-1 **(H)**. β-actin was used as loading control. The intensities of the bands were expressed as the arbitrary units. *P < 0.05. Bars that do not share the same letter are significantly different (*P* < 0.05).

**Figure 7:**
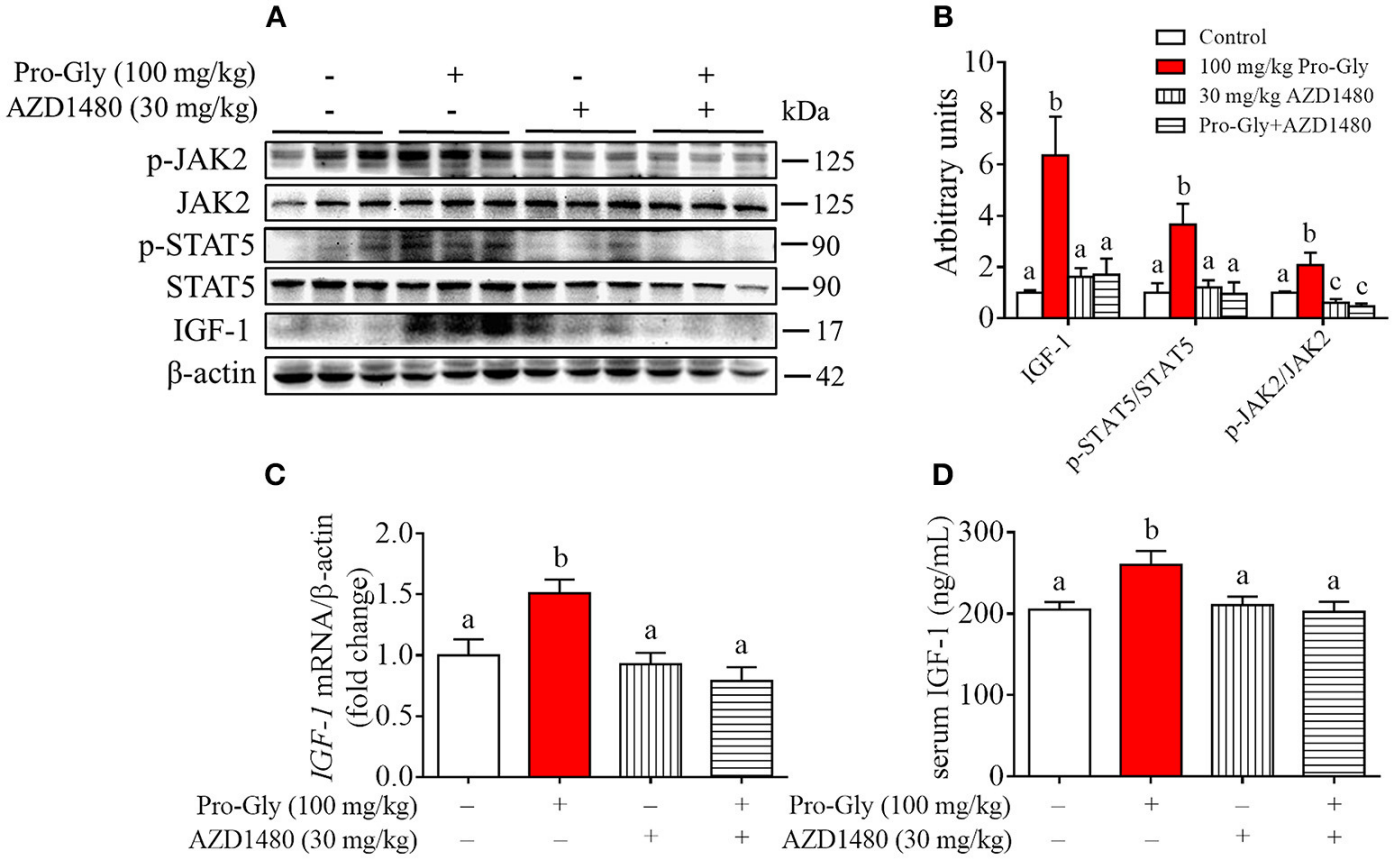
Injection of JAK2/STAT5 inhibitor abolished the promotive effect of Pro-Gly on IGF-1 expression and secretion in mice. Twenty-eight 6-week old female mice were randomly divided into 4 groups (*n* = 7). **(A)** Western blot analysis of phospho-JAK2 (p-JAK2), JAK2, phospho-STAT5 (p-STAT5), STAT5 and prepro IGF-1 in mice liver after injection. β-actin was used as loading control. The mice were intraperitoneal injected with physiological saline (Control), Pro-Gly (100 mg/kg, 1 h), JAK2 inhibitor AZD1480 (30 mg/kg, 3 h) or Pro-Gly+AZD1480 in a volume of 100 μL, respectively. **(B)** Mean ± SEM of immunoblotting bands of prepro IGF-1, p-JAK2/JAK2, and p-STAT5/STAT5 (*n* = 7). The intensities of the bands were expressed as the arbitrary units. **(C)** *IGF-1* mRNA level in mice liver (*n* = 7). **(D)** Serum levels of IGF-1 (*n* = 7). Bars that do not share the same letter are significantly different (*P* < 0.05).

Western blot assay was conducted as we previously described (35). Briefly, the HepG2 cells or liver tissue were lysed in RIPA lysis buffer [25 mM Tris•HCl (pH 7.6), 150 mM NaCl, 1% NP-40, 1% sodium deoxycholate, and 0.1% SDS] containing 1 mM PMSF. The total protein concentration was determined using BCA protein assays. After separation on 10% SDS-PAGE gels, the proteins were transferred electrophoretically to polyvinylidene fluoride (PVDF) membranes, and then blocked with 6% (wt/vol) non-fat dry milk in Tris-buffered saline (TBS) containing 0.1% Tween 20 for 2 h at room temperature. Subsequently, the PVDF membranes were exposed for overnight at 4°C to the primary antibodies, including β-actin (1:2,000), β-tubulin (1:2,000), IGF-1 (prepro peptide with the MW of 17 kDa, 1:1,000), JAK2 (1:1,000), phospho-JAK2 (1:1,000), STAT5 (1:1,000), and phospho-STAT5 (1:1,000). The membrane was washed with TBS containing 0.1% Tween 20, incubated for 1 h at room temperature with appropriate secondary antibodies (Bioworld, Nanjing, China) and washed again with TBS containing 0.1% Tween 20. Then, the PVDF membrane were incubated with EMD Millipore™ Immobilon™ Western Chemiluminescent HRP Substrate (ECL) (Millipore, Burlington, USA) and immunoreactive proteins in the membrane were scanned with a FluorChemMFluorescent Imaging System (ProteinSimple, Santa Clara, CA, USA). The band density was normalized according to the β-actin or β-tubulin expression. The raw bands of Figures 1–**7** were provided in Data Sheet 1.

### Radioimmunoassay

IGF-1 radioimmunoassay kit was purchased from Jiuding Medical Biological Engineering Co., Ltd. (Tianjin, China). Mice serum and cell culture supernatant IGF-1 concentration were measured by GC-1200 Gamma RIA counter (Zhongke zhongjia Instruments, Inc., Anhui, China) according to the manufacturer's recommendation.

### Co-immunoprecipitation

Lysates containing 500 μg total protein were immunoprecipitated with antibodies specific to JAK2 overnight at 4°C. Immune complexes were collected by incubation with a mixture of protein A- and G-Sepharose (Beyotime Biotechnology, Shanghai, China) for 6 h at 4°C; the immune complexes were then washed three times with wash buffer [50 mM HEPES-NaOH (pH 7.6), 150 mM NaCl, and 0.1% Triton X-100] before being eluted in 2 × sodium dodecyl sulfate sample buffer. The immune complexes were subjected to SDS-PAGE and transferred to a polyvinylidene difluoride membrane for further protein detection.

### P-STAT5 translocation

Cell climbing slices were rinsed 3 times in PBS, fixed in paraformaldehyde for 10 min and washed in 0.4% Triton X-100 (Sigma) for 30 min then blocked for 1 h at room temperature. Subsequently, the slices were incubated overnight in rabbit anti-phospho-STAT5 (Abcam) at 4°C. The next day, the slices were incubated in FITC second antibody (Bioss) for 1 h and then incubated in DAPI (Bioss) for 10 min. HepG2 cells were then observed and the fluorescence were quantified using Nikon Eclipse Ti-s microscopy with Nis Elements BR software (Nikon Instruments, Japan). Up to six fields of view were captured from every group.

### Transfection of HepG2 with PepT1 siRNA

The HepG2 cells were transfected with 4 pmol of siRNA specific for PepT1 (GenePharma Co., Ltd, Shanghai, China) or scrambled siRNA using Lipofectamine 2000 (Invitrogen, Carlsbad, CA, USA) for 6 h according to the manufacturer's instructions. Subsequently, the cells were treated with Pro-Gly for 24 h. The knockdown efficiency of PepT1 was confirmed by qPCR.

### Statistical analysis

Data are presented as means ± standard error of the mean (SEM). For the cell culture studies, the experiments were independently repeated at least 3 times, with 6 replicates each time. In the mouse feeding trial, individual animal was considered as an experimental unit and 6–10 mice in each group were used. Differences between means were determined using Student's *t*-test or one-way analysis of variance (ANOVA) followed by *post-hoc* Tukey test when appropriate and a confidence level of *P* < 0.05 was considered to be statistically significant (SPSS 20.0, Chicago, IL, USA). All graphs were plotted with GraphPad Prism 6.01 (GraphPad software, San Diego, CA, USA).

## Results

### Pro-gly promoted the expression and secretion of IGF-1 in the HepG2 cells

In order to investigate the effects of Pro-Gly on IGF-1 expression and secretion, the HepG2 cells were incubated for 24 h in the presence of 0.2, 0.5, and 1 mM Pro-Gly. The *IGF-1* mRNA level in HepG2 cells was significantly (*P* < 0.05) elevated by Pro-Gly (0.5 mM; Figure 1A), which had no effect on cell viability of HepG2 cells (see Figure S1 in Data Sheet 1). Consistently, the protein expression of prepro IGF-1 in HepG2 cells was markedly (*P* < 0.05) enhanced by Pro-Gly (0.5 and 1 mM; Figures 1B,C). In addition, the results of RIA for IGF-1 showed that Pro-Gly (0.2, 0.5, and 1 mM) significantly (*P* < 0.05) increased the IGF-1 levels in the cell culture supernatant (Figure 1D).

### Pro-gly, but not pro plus gly, promoted the expression and secretion of IGF-1 in the HepG2 cells

To elucidate whether the effects of Pro-Gly on IGF-1 expression and secretion were attributed to its degradation products Pro and Gly, we incubated the HepG2 cells with Pro-Gly or Pro plus Gly (Pro+Gly). As shown in Figure 2, in contrast to the promotion of IGF-1 expression and secretion induced by Pro-Gly, the combination of Pro and Gly (Pro+Gly) had no effect on IGF-1, suggesting that Pro-Gly elicited its effects on IGF-1 expression and secretion by itself but not its degradation products.

### Knockdown of PepT1 eliminated the promotion of IGF-1 expression induced by pro-gly in the HepG2 cells

To determine the possible role of the peptide transporter PepT1 in Pro-Gly-promoted IGF-1 expression and secretion in hepatocytes, we first examined the expression of PepT1 in response to Pro-Gly in HepG2 cells and found that the dipeptide Pro-Gly was able to increase the *PepT1* mRNA level in the HepG2 cells (Figure 3A). Further, PepT1 siRNA were used to knockdown PepT1, and the result demonstrated that PepT1 siRNA significantly decreased the *PepT1* mRNA level, with no difference between the group of no-siRNA control and scrambled siRNA (Figure 3B). In addition, the increase of *PepT1* mRNA level induced by Pro-Gly was eliminated in the presence of PepT1 siRNA (Figure 3A). Accordingly, the promotion of IGF-1 expression induced by Pro-Gly was abolished by PepT1 knockdown with PepT1 siRNA (Figures 3C,D). Together, these observations showed that Pro-Gly-stimulated IGF-1 expression in HepG2 cells was mediated, at least in part, through PepT1.

### Pro-gly activated JAK2/STAT5 signaling pathway in a PepT1-dependent manner

We further investigated the intracellular signaling pathways which were likely involved in the Pro-Gly-promoted IGF-1 secretion in HepG2 cells. The Western blot findings revealed that the ratio of p-JAK2/JAK2 was significantly (*P* < 0.05) enhanced by Pro-Gly (0.5 mM), indicating the activation of JAK2 signal pathway (Figures 4A,B). Consistently, STAT5, a downstream target of JAK2, was also activated by Pro-Gly, with a significant (*P* < 0.05) increase of p-STAT5/STAT5 ratio. In other words, Pro-Gly activated JAK2/STAT5 signaling pathway in HepG2 cells. Interestingly, the activation of JAK2/STAT5 signaling pathway was reversed by PepT1 knockdown with PepT1 siRNA (Figures 4A,B), suggesting that Pro-Gly activated JAK2/STAT5 signaling pathway in a PepT1-dependent manner.

Results from Co-IP experiments showed that the interaction between JAK2 and STAT5 was enhanced by Pro-Gly treatment in HepG2 cells (Figure 4C). Consistently, Pro-Gly treatment increased phospho-STAT5 translocation to nuclei in HepG2 cells (Figure 4D and Figure S3 in Data Sheet 1). These findings further suggested that activation of the JAK2/STAT5 signaling pathway might contribute to Pro-Gly-increased IGF-1 expression and secretion in HepG2 cells through PepT1.

### Inhibition of JAK2/STAT5 signaling pathway blocked the promotive effect of pro-gly on IGF-1 expression and secretion in the HepG2 cells

To further verify the role of JAK2/STAT5 signaling pathway in Pro-Gly-promoted IGF-1 expression and secretion in the HepG2 cells, AZD1480, a specific and potent inhibitor of JAK2, was applied in the present study. As shown in Figures 5A,B, the elevated ratios of p-JAK2/JAK2 and p-STAT5/STAT5 induced by Pro-Gly were abolished by the inhibition of JAK2 with AZD1480 (Figures 5A,B). Meanwhile, the increased intranuclear p-STAT5 level in response to Pro-Gly was eliminated by the inhibition of JAK2 with AZD1480, which alone had no effect on the level of nuclear p-STAT5 (Figure 4D). In addition, the Pro-Gly-induced increase in IGF-1 mRNA and protein expression in HepG2 cells was also blocked by AZD1480 (Figures 5A–C). Moreover, the RIA result of IGF-1 indicated that AZD1480 alone had no effect on the IGF-1 level in cell culture supernatant. However, AZD1480 could eliminate the promotive effect of Pro-Gly on IGF-1 secretion (Figure 5D). These results strongly suggested that Pro-Gly stimulated IGF-1 expression and secretion through activating JAK2/STAT5 signaling pathway.

### Acute or chronic injection of pro-gly, but not pro plus gly, stimulated IGF-1 expression and secretion in the mice

We also determined the *in vivo* effect of Pro-Gly on IGF-1 expression and secretion by acute or chronic injection of Pro-Gly or Pro plus Gly (Pro+Gly) in C57BL/6J mice. As shown in Figures 6A–D, in contrast to the increase of mRNA and protein expression in mice liver and serum levels of IGF-1 induced by acute injection of Pro-Gly, the combination of Pro and Gly (Pro+Gly) had no influence on IGF-1 expression and secretion. In addition, the Western blot findings revealed that the JAK2/STAT5 signal pathway was activated by Pro-Gly but not Pro plus Gly (Pro+Gly) in mice liver (Figures 6B,C). Similar to the results of acute injection experiment, chronic injection of Pro-Gly, but not Pro plus Gly (Pro+Gly), promoted IGF-1 expression and secretion and activated JAK2/STAT5 signal pathway in the mice liver (Figures 6F–H). It should be noted that chronic injection of Pro-Gly had no liver toxicity in vivo (see Figure S2 in Data Sheet 1). Furthermore, we also examined the expression of PepT1 in the liver of C57BL/6J mice injected with Pro-Gly chronically. The results demonstrated that the dipeptide Pro-Gly increased the *PepT1* mRNA level in the mice liver, suggesting the potential role of PepT1 in the Pro-Gly-stimulated IGF-1expression and secretion (Figure 6E).

### Acute injection of JAK2/STAT5 inhibitor abolished the promotive effect of pro-gly on IGF-1 expression and secretion in mice

To further verify the role of JAK2/STAT5 signaling pathway in Pro-Gly-promoted IGF-1 expression and secretion in mice, AZD1480, a specific and potent inhibitor of JAK2, was applied in the acute injection experiment. As expected, the elevated ratios of p-JAK2/JAK2 and p-STAT5/STAT5 induced by Pro-Gly were abolished by the inhibition of JAK2 with AZD1480, which alone had no significant effect on ratios of p-JAK2/JAK2 and p-STAT5/STAT5 (Figures 7A,B). In agreement, the increase of IGF-1 mRNA and protein expression in liver induced by Pro-Gly was also abrogated by AZD1480 (Figures 7A–C). In addition, the RIA result of IGF-1 indicated that AZD1480 alone had no effect on the serum level of IGF-1 in mice. However, AZD1480 eliminated the promotive effect of Pro-Gly on IGF-1 secretion (Figure 7D). These results strongly suggested that Pro-Gly stimulated IGF-1 expression and secretion through enhancing JAK2/STAT5 signaling pathway *in vivo*.

## Discussion

In this paper, we investigated the effects of the dipeptide Pro-Gly on IGF-1 expression and secretion in HepG2 cells and C57BL/6J mice and the signaling pathways underlying this process. Our results demonstrated that the dipeptide Pro-Gly promoted IGF-1 expression and secretion by enhancing JAK2/STAT5 signaling pathway through PepT1. We previously reported that the dipeptide Pro-Asp promotes IGF-1 secretion and expression in HepG2 cells and porcine hepatocytes (36). Similarly, in the present study, we found that the dipeptide Pro-Gly was able to promote IGF-1 expression and secretion in the HepG2 cells. Interestingly, the stimulation of Pro-Gly on IGF-1 expression and secretion was not observed when HepG2 cells were incubated with Pro plus Gly (Pro+Gly), suggesting the particular role of Pro-Gly but not of the related individual amino acid. In addition, *in vivo* studies were conducted to further address the biological effects of Pro-Gly on IGF-1 expression and secretion. Similar to the *in vitro* results, acute injection of Pro-Gly, but not Pro plus Gly, increased IGF-1 protein expression in mice liver and serum IGF-1 level. Furthermore, it was reported that the physiological importance of oligopeptides became apparent when their luminal concentrations were much higher than that of free amino acids after a controlled protein diet in man (37). Together, our findings showed that Pro-Gly, but not Pro plus Gly, promoted the expression and secretion of IGF-1 in HepG2 cells and C57BL/6J mice.

It has been reported that PepT1, a transporter for di-or tri-peptides, is involved in regulating various biological processes or functions, including the uptake of the dipeptide Gly-Sar in mice jejunum (38), the anti-oxidative stress of the dipeptide Ala-Gln in caco-2 cells (39), and the proinflammatory response of the tripeptide L-Ala-γ-D-Glu-*meso*-DAP (Tri-DAP) in intestinal epithelial cells (40). To elucidate the possible function of PepT1 in Pro-Gly-stimulated IGF-1 expression and secretion in hepatocytes, we examined the mRNA level of *PepT1* in HepG2 cells and mice liver. Similar to the previous result (41), we found that *PepT1* was expressed in HepG2 cells and mice liver. More importantly, our results demonstrated that *PepT1* mRNA level was significantly increased by the dipeptide Pro-Gly. Furthermore, PepT1 knockdown with PepT1 siRNA reversed the Pro-Gly-promoted IGF-1 expression and secretion of HepG2 cells. Together, these observations showed that Pro-Gly-stimulated IGF-1 expression and secretion of HepG2 was mediated, at least in part, through PepT1.

Many studies have shown that the activation of JAK2/STAT5 signaling pathway is involved in promoting IGF-1 secretion (42–44). In line with the previous reports, we found that the JAK2/STAT5 signaling pathway was activated during the process of Pro-Gly-promoted IGF-1 expression and secretion in HepG2 cells and mice liver. In addition, the activation of JAK2/STAT5 signaling pathway induced by Pro-Gly was abolished by PepT1 knockdown. These results suggested that Pro-Gly activated JAK2/STAT5 signaling pathway in a PepT1-dependent manner. However, it has also been demonstrated that JAK2 is able to regulate multiple cellular carriers including PepT1 (45). The inconsistent upstream or downstream regulation between PepT1 and JAK2/STAT5 signaling pathway might be due to the different culture conditions such as cell types, culture systems and treatment times. It has been reported that activation of JAK/STAT5 signaling pathway is accompanied with the interaction between JAK2 and STAT5, and the translocation of STAT5 to the nuclei, thus contributing to hepatic IGF-1 expression and secretion induced by arginine treatment in HepG2 cells (46). In agreement, our results showed that Pro-Gly treatment increased the interaction between JAK2 and STAT5 and the translocation of phospho-STAT5 to the nuclei in HepG2 cells. These findings implied that the activation of the JAK2/STAT5 signaling pathway might contribute to Pro-Gly-increased IGF-1 expression and secretion in HepG2 cells through PepT1. However, in our previous report (36), we did not know the plasma membrane mechanism of Pro-Asp-promoted IGF-1 production.

We further verify the role of JAK2/STAT5 signaling pathway in Pro-Gly-promoted IGF-1 expression and secretion by inhibiting JAK2/STAT5 signaling pathway *in vitro* and *in vivo*. Our results demonstrated that the inhibition of JAK2 with AZD1480 totally reversed the increase of IGF-1 expression and secretion, and translocation of p-STAT5 to the nuclei in HepG2 cells, which were induced by Pro-Gly. In addition, the *in vivo* study also revealed that the Pro-Gly-induced increase of IGF-1 mRNA and protein expression in liver and elevation of the serum level of IGF-1 in mice was abolished by the inhibition of JAK2 with AZD1480. Similarly, we previously reported that inhibition of JAK2/STAT5 signaling pathway eliminated the Pro-Asp-stimulated IGF-1 expression and secretion in hepatocytes (36). These results strongly suggested that JAK2/STAT5 signaling pathway was involved in the promotive effects of Pro-Gly on IGF-1 expression and secretion in HepG2 cells and C57BL/6J mice.

In conclusion, our results demonstrated that, an absorbable and bioactive dipeptide Pro-Gly, promoted IGF-1 expression and secretion in HepG2 cells and C57BL/6J mice by activating JAK2/STAT5 signaling pathway, which is mediated via the PepT1. These data provided new insights to the regulation of IGF-1 expression and secretion by the dipeptides.

## Author contributions

MZ, JX, TW, XW, and FZ performed experiments. All authors contributed to discussion and review of the manuscript. SW and QJ designed the experiments. SW and MZ analyzed data, did the interpretation, and wrote the manuscript.

### Conflict of interest statement

The authors declare that the research was conducted in the absence of any commercial or financial relationships that could be construed as a potential conflict of interest.

## Abbreviations

GH: growth hormone
Gly: glycine
IGF-1: insulin-like growth factor 1
JAK2: Janus kinase 2
PepT1: proton-coupled peptide transporter 1
Pro: proline
RIA: radioimmunoassay
STAT5: signal transducer and activator of transcription 5.
