# Supplementary material for: The Dipeptide Pro-Gly Promotes IGF-1 Expression and Secretion in HepG2 and Female Mice via PepT1-JAK2/STAT5 Pathway
Source: Front Endocrinol (Lausanne). 2018 Jul 26;9:424. doi: 10.3389/fendo.2018.00424 (PMC6094964; doi:10.3389/fendo.2018.00424)

**Supplementary data**

Supplemental Figures

Fig. S1 Effect of Pro-Gly on the cell viability in vitro. Cell counting kit-8 assay was used to examine HepG2 cells viability after 24 h incubation with 0.5 mM Pro-Gly (n=6). Pro-Gly had no effect on cell viability in HepG2 cells. Data are presented as mean±SEM.

Fig. S2 Effects of chronic injection of Pro-Gly on *ALT、OTC* and *SDH* mRNA levels. The 30 four-week-old female mice were intraperitoneal injected with physiological saline (Control, n=10) or Pro-Gly (150 mg/kg, n=10) every other day for 35 days. Pro-Gly had no significant effects on the *ALT*, *OTC* and *SDH* mRNA levels compared with control group, suggesting that injection of Pro-Gly had no effect on liver toxicity *in vivo*. Data are presented as mean±SEM.

Fig. S3 Effect of Pro-Gly on the relative fluorescence intensity of p-STAT5 in nuclei of HepG2 cells. HepG2 cells were incubated in the presence of Pro-Gly (0.5 mM) and/or AZD1480 (1 μM) for 6 h and phospho-STAT5 translocation to nuclei was detected by ICC. The fluorescence intensity was quantified with Nis-Elements BR software (Nikon Instruments, Tokyo, Japan) and the mean relative fluorescent intensity (MRFI) from the nucleus was analyzed (Control: n=19, Pro-Gly: n=23, AZD1480: n=26, Pro-Gly+AZD1480: n=29). Pro-Gly treatment increased the relative fluorescence intensity of phospho-STAT5 in HepG2 cells nuclei. However, the increased intranuclear p-STAT5 level in response to Pro-Gly was eliminated in the presence of JAK2 inhibitor AZD1480, which alone had no effect on the level of nuclear p-STAT5*.* Data are presented as mean ± SEM. Bars that do not share the same letter are significantly different (*P* < 0.05).

Images used for fluorescence intensity analysis for Fig. S3.

Control


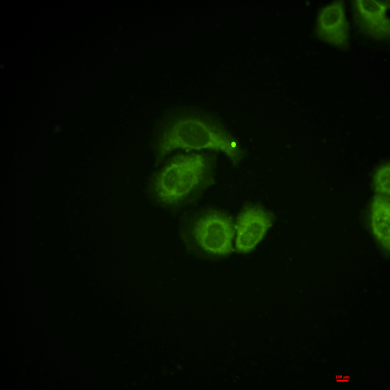

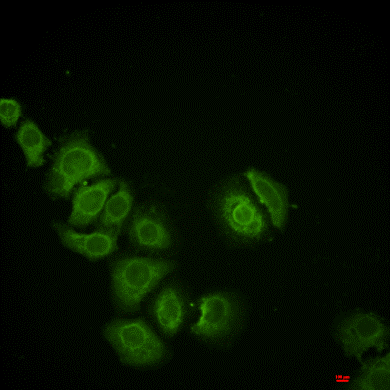


Pro-Gly


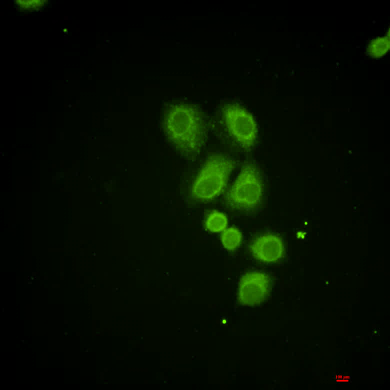

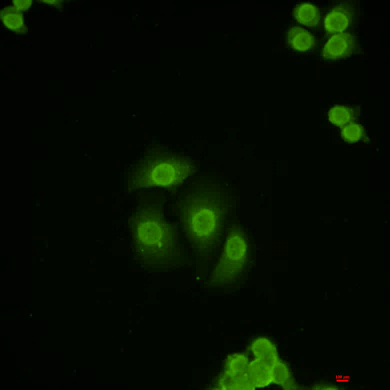


AZD1480


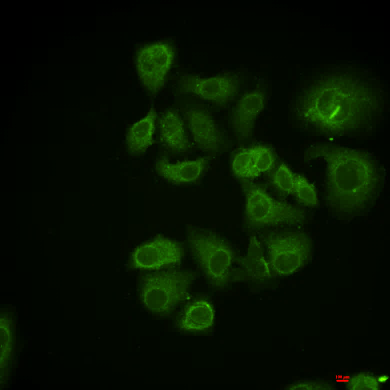

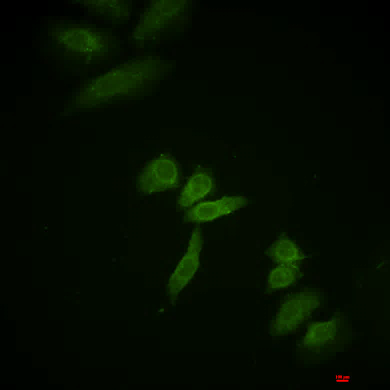


Pro-Gly+AZD1480


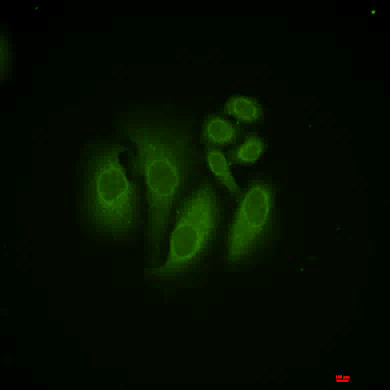

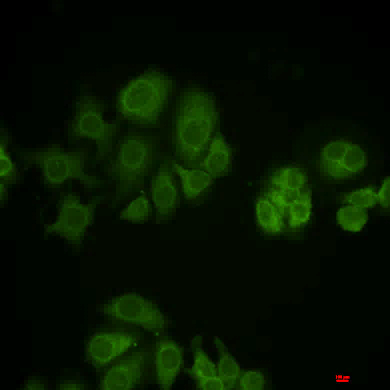


The raw bands of Western blot

Figure 1

IGF-1


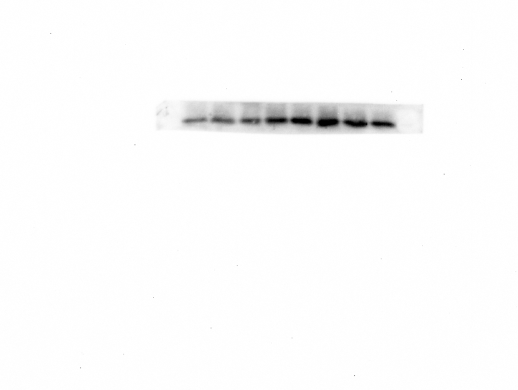


β-tubulin


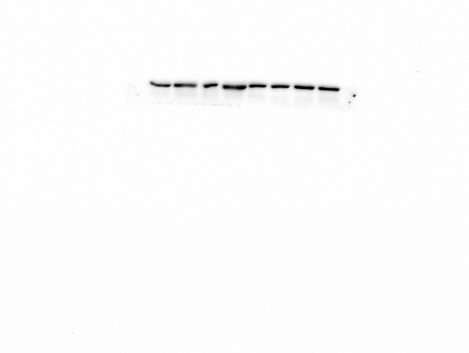


Figure 2

IGF-1


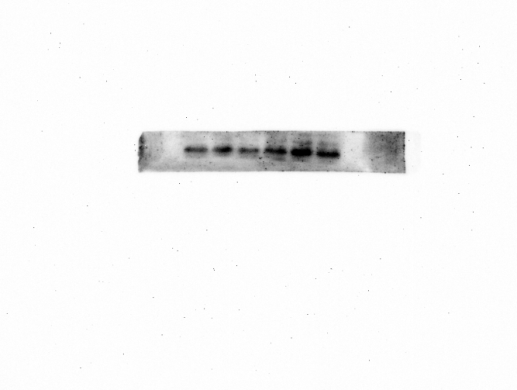


β-actin (the last six)


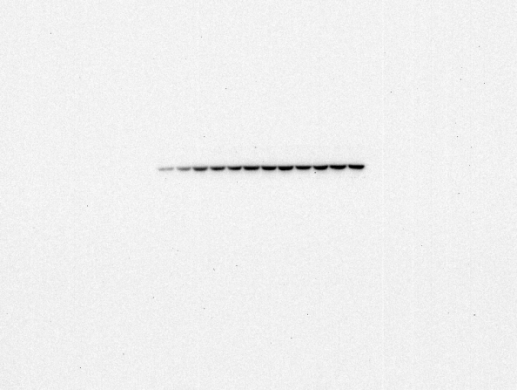


Figure 3

IGF-1


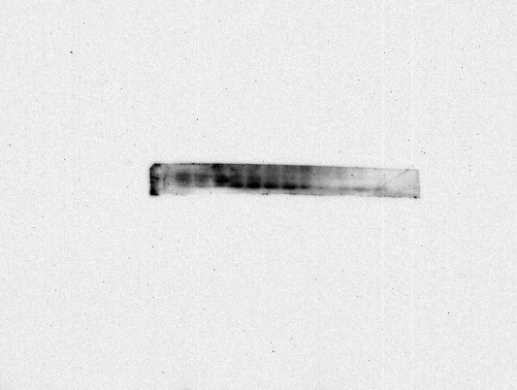


β-actin


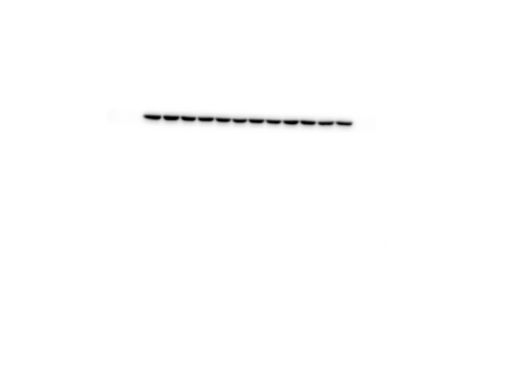


Figure 4A

p-JAK2


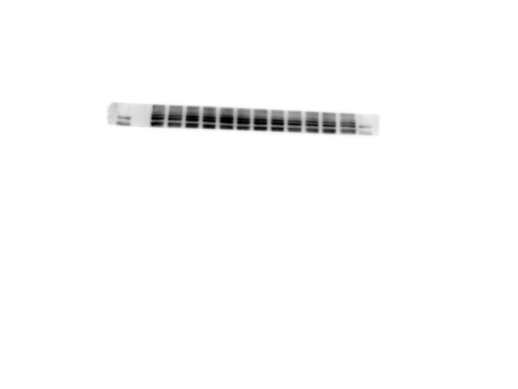


JAK2


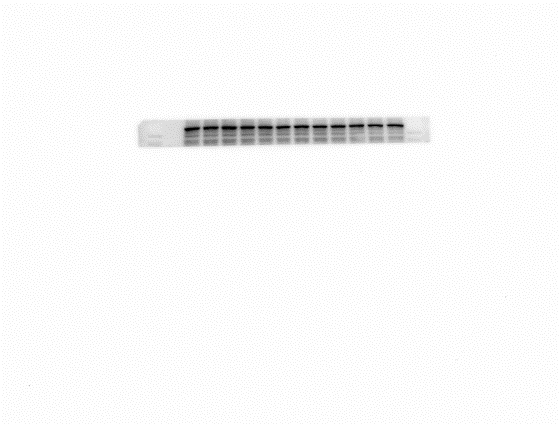


p-STAT5


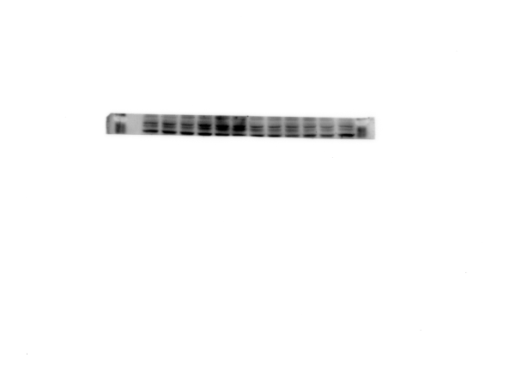


STAT5


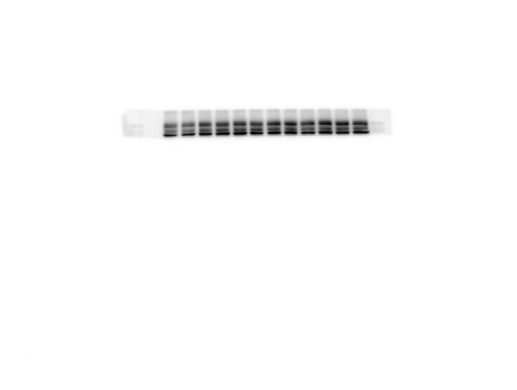


β-actin


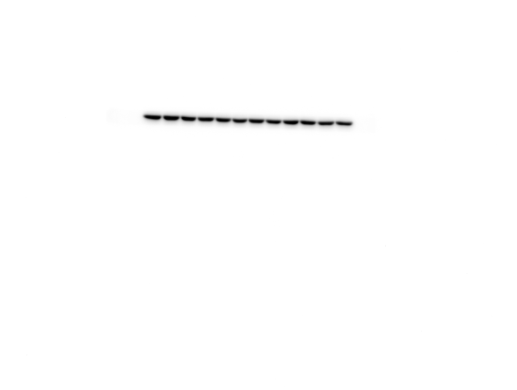


Figure 4C

IP: STAT5


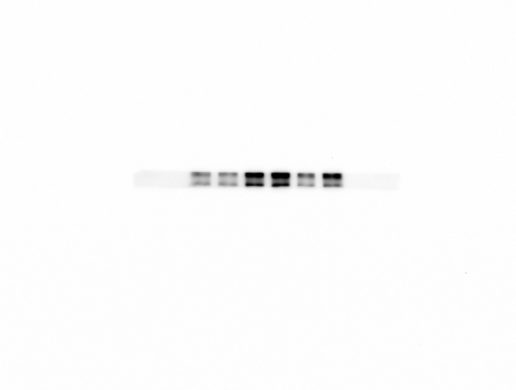


IP: JAK2


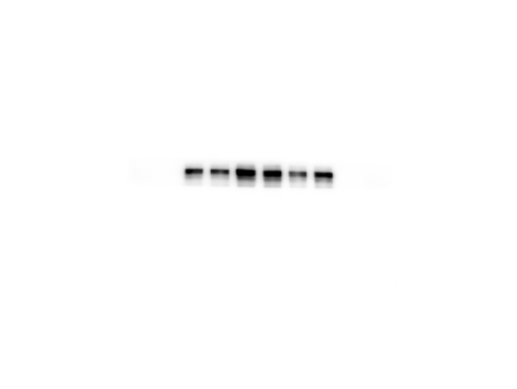


Input: STAT5


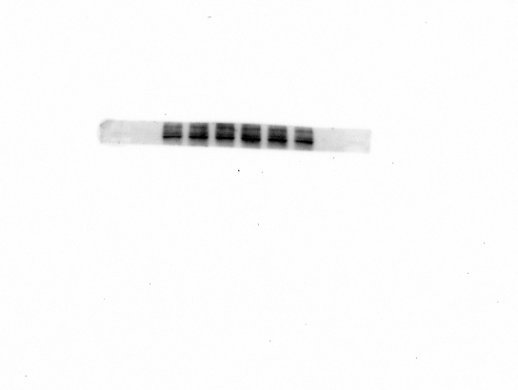


Input: JAK2


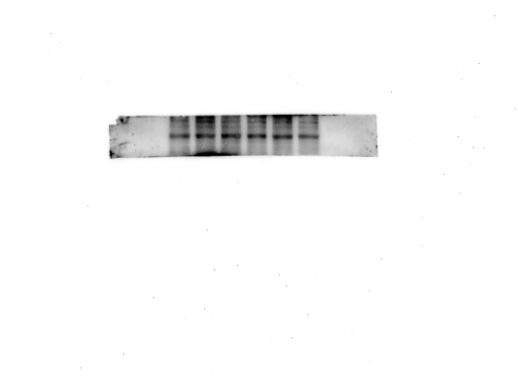


β-actin


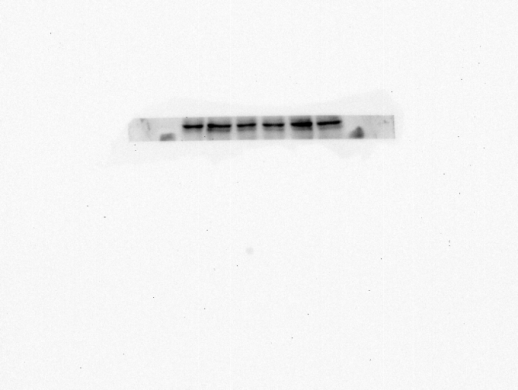


Figure 5

p-JAK2


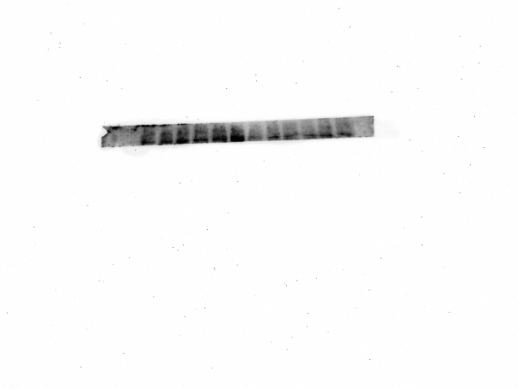


JAK2


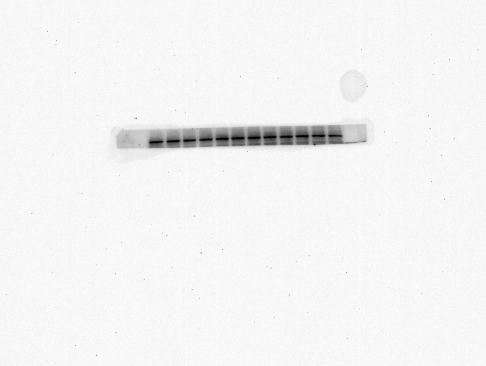


p-STAT5


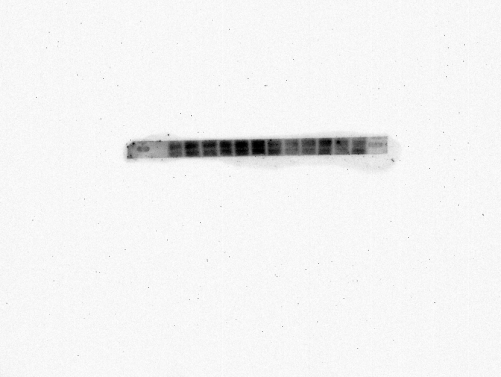


STAT5


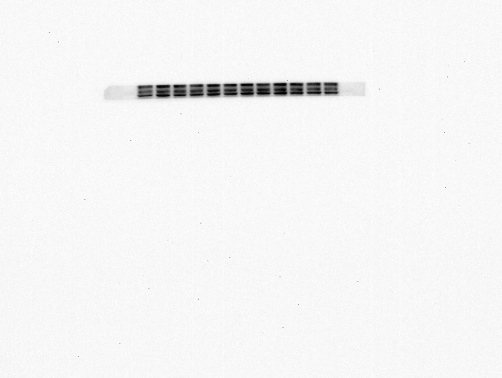


β-actin


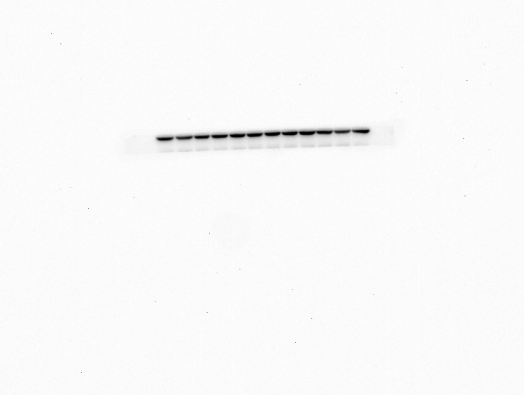


IGF-1


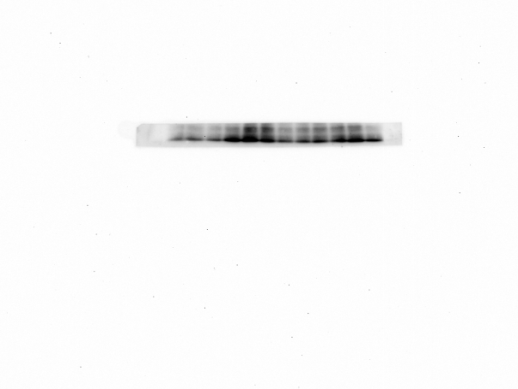

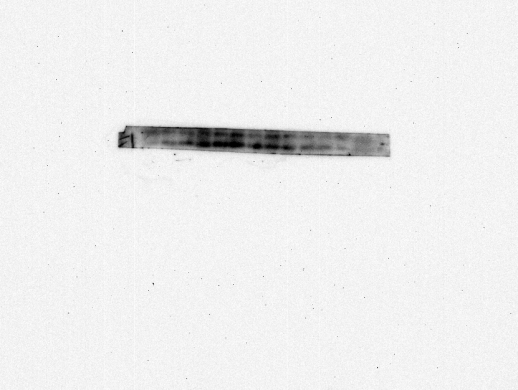


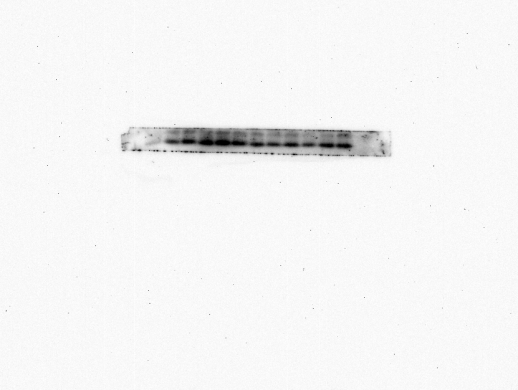

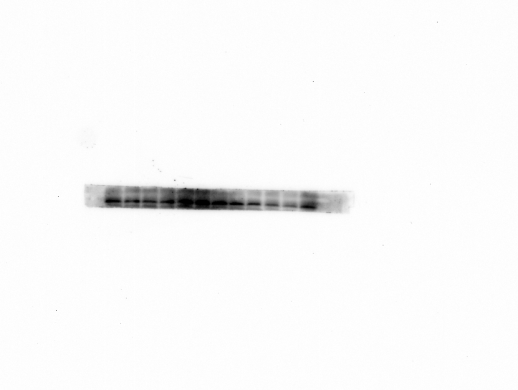


β-actin


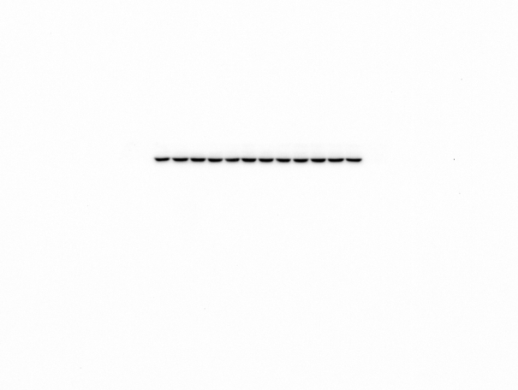


Figure 6B

p-JAK2 (the first six)


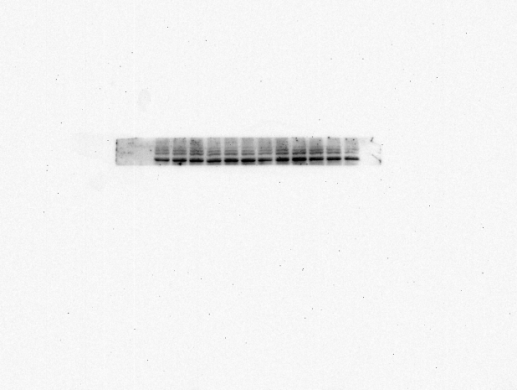


JAK2 (the first six)


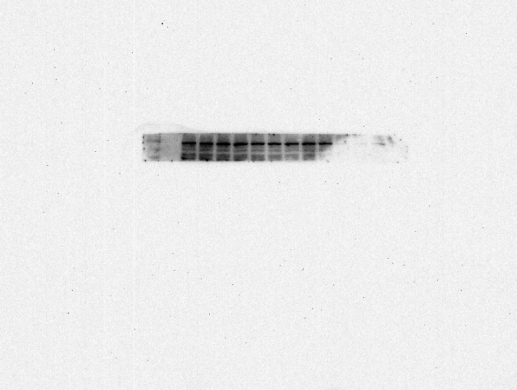


p-STAT5


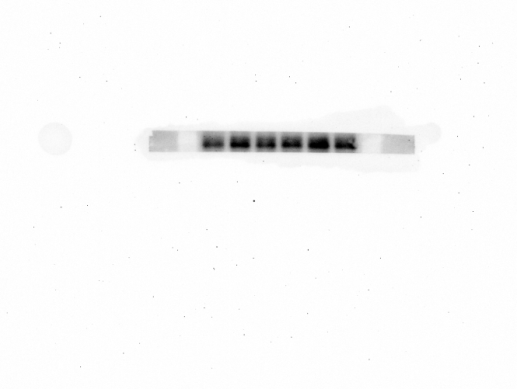


STAT5


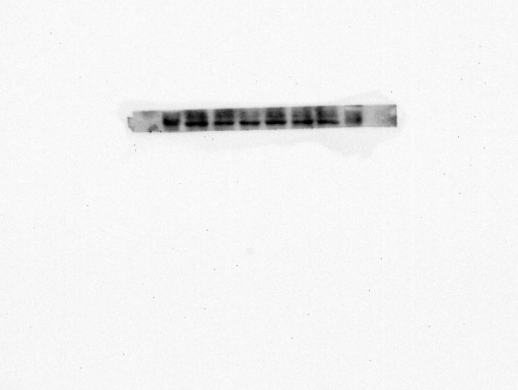


IGF-1


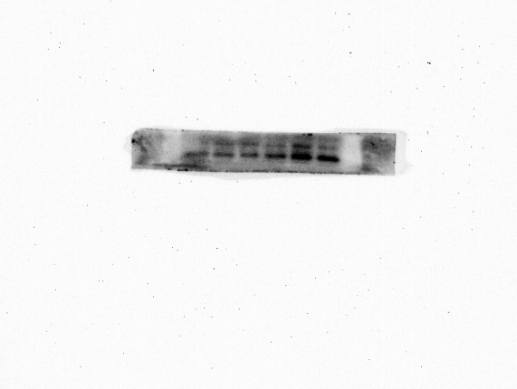


β-actin


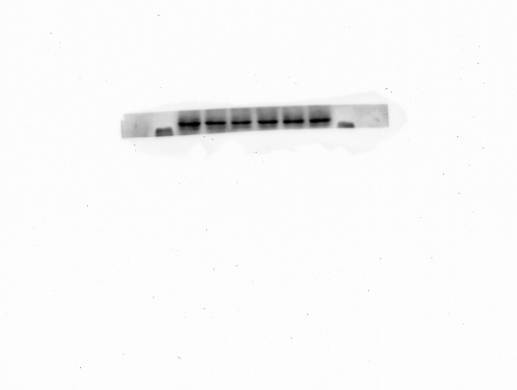


Figure 6F

p-JAK2


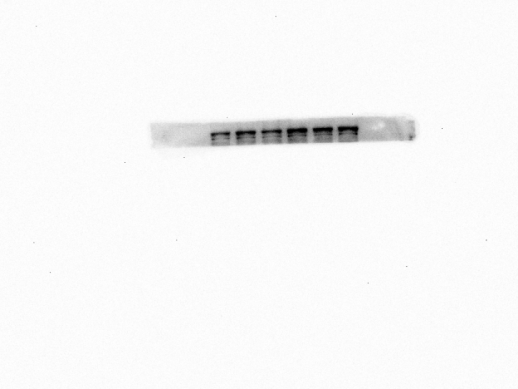


JAK2


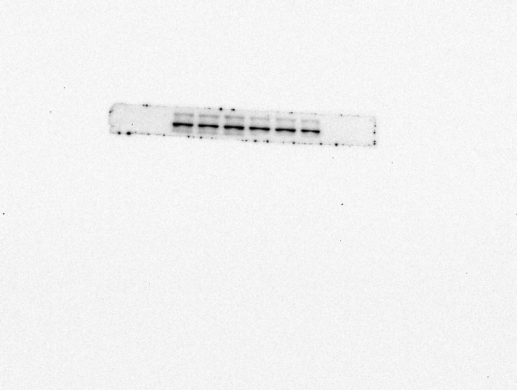


p-STAT5


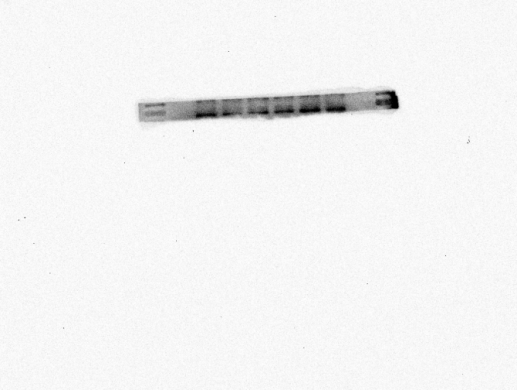


STAT5


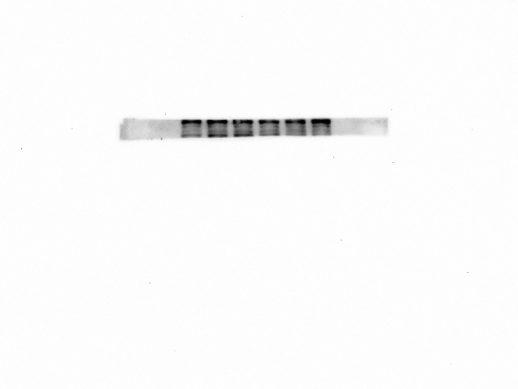


IGF-1


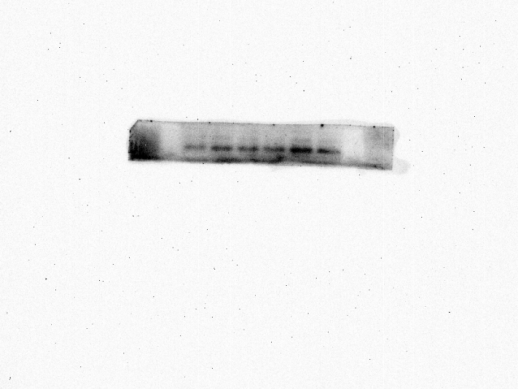


β-actin


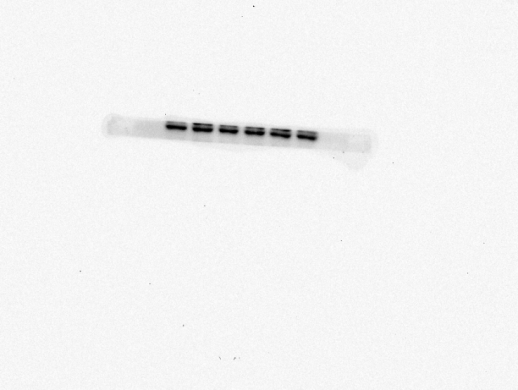


Figure 7

p-JAK2


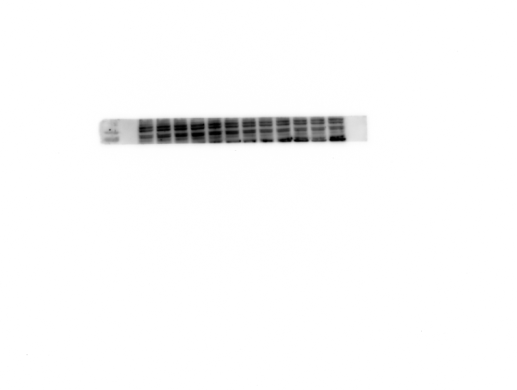


JAK2


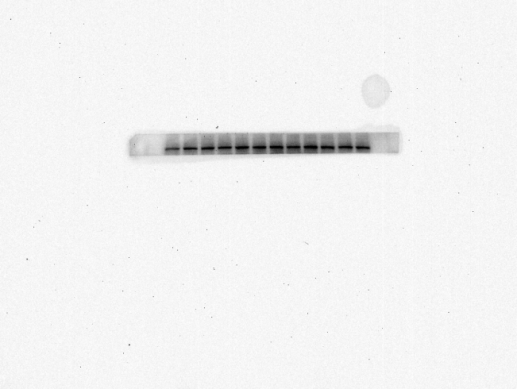


p-STAT5


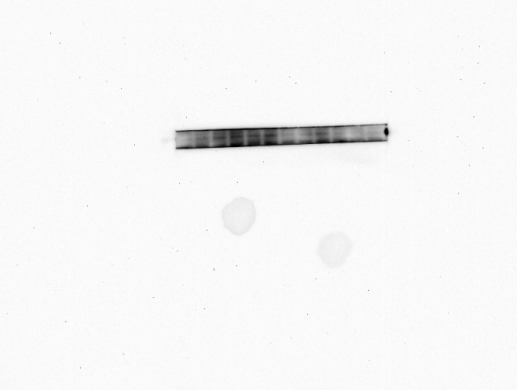


STAT5


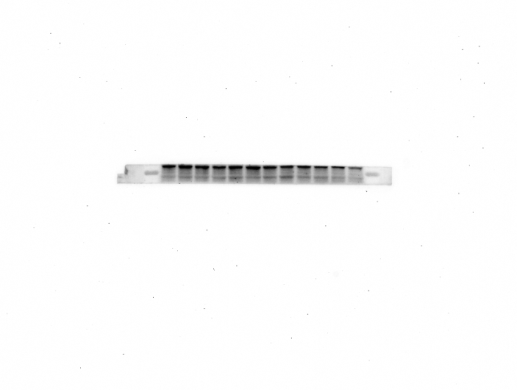


IGF-1


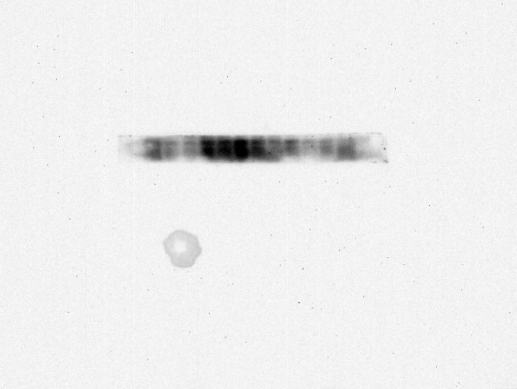


β-actin


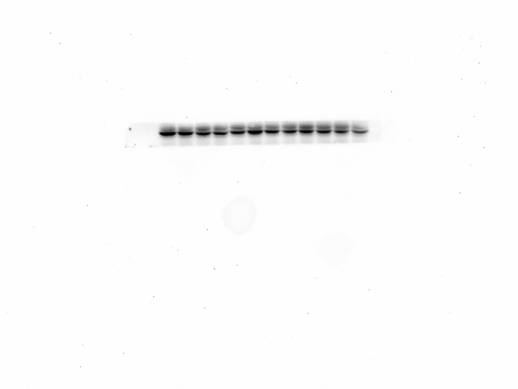

Supplement: Supplementary file 1 [file Data_Sheet_1.docx]
